# D-Limonene Inhibits *Pichia kluyveri* Y-11519 in Sichuan Pickles by Disrupting Metabolism

**DOI:** 10.3390/molecules29153561

**Published:** 2024-07-28

**Authors:** Chaoyi Zeng, Yue Sun, Haoran Lin, Ziyu Li, Qing Zhang, Ting Cai, Wenliang Xiang, Jie Tang, Patchanee Yasurin

**Affiliations:** 1 College of Food and Bioengineering, Xihua University, Chengdu 610039, China; 17780770409@163.com (Y.S.); 212022095100016@stu.xhu.edu.cn (H.L.); catalpa_123@163.com (Z.L.); caiting1124@mail.xhu.edu.cn (T.C.); biounicom@mail.xhu.edu.cn (W.X.); tangjie1225@mail.xhu.edu.cn (J.T.); 2 Department of Food Biotechnology, Faculty of Biotechnology, Assumption University, Bangkok 10240, Thailand; patchaneeysr@au.edu; 3 Food Microbiology Key Laboratory of Sichuan Province, Xihua University, Chengdu 610039, China

**Keywords:** *Pichia kluyveri*, D-limonene, cell wall and membrane synthesis, oxidative stress, glycolysis and tricarboxylic acid cycle

## Abstract

The *Pichia kluyveri*, a proliferation commonly found in Sichuan pickles (SCPs), can accelerate the growth and reproduction of spoilage bacteria, causing off-odor development and decay. Although D-limonene, a common natural preservative, effectively restricts *P. kluyveri*, its inhibitory mechanism remains unclear. This study aimed to elucidate this molecular mechanism by investigating the impact on basic *P. kluyveri* metabolism. The findings revealed that D-limonene inhibited *P. kluyveri* growth and disrupted the transcription of the genes responsible for encoding the enzymes involved in cell wall and membrane synthesis, oxidative phosphorylation, glycolysis, and the tricarboxylic acid (TCA) cycle pathway. The results indicated that these events disrupted crucial metabolism such as cell wall and membrane integrity, adenosine triphosphate (ATP) synthesis, and reactive oxygen species (ROS) balance. These insights provided a comprehensive understanding of the inhibitory effect of D-limonene on the growth and reproduction of *P. kluyveri* while highlighting its potential application in the SCP industry.

## 1. Introduction

Sichuan pickles (SCPs) are a unique fermented vegetable product from the Sichuan region and a representative of Chinese food culture. They can be produced on both household and industrial scales. With their unique flavor and rich taste, Sichuan pickles are highly favored by consumers [1,2,3]. Traditionally, Sichuan pickles are naturally fermented through the competition between indigenous lactic acid bacteria in old brine and microorganisms in fresh vegetables [3]. During fermentation, the microbial community is easily influenced by the raw vegetables and the fermentation environment [4,5].

Among these microorganisms, *Pichia kluyveri* is commonly found in nature, especially in soil, plant surfaces, rotting fruit, and fermented foods [6,7,8]. It tolerates acidic environments and high osmotic pressure [9,10], easily enters the fermentation environment through fresh vegetables, competes with lactic acid bacteria for nutrients, inhibits their growth, and reduces lactic acid production, affecting the fermentation outcome. As fermentation progresses, *P. kluyveri* forms a membrane at the air–liquid interface, which is considered a sign of quality deterioration in fermented vegetables [3]. Arroyo et al. isolated *Pichia kluyveri* from spoiled fermented olives using turbid brine, demonstrating its role in film formation [11]. Cai et al. identified *Pichia kluyveri* SH2 and *Candida tropicalis* SH1 as representative microorganisms responsible for film formation in Sichuan pickles [12]. Kim et al. found that *Pichia kluyveri* is one of the microorganisms associated with the spoilage of Korean kimchi [13]. These studies confirm that *Pichia kluyveri* forms a membrane, isolating the saline environment, alleviating pH stress, and facilitating the growth of spoilage microorganisms, consequently expediting SCP tissue softening [1,14]. Therefore, it is crucial to explore effective methods for controlling *P. kluyveri* to prevent SCP spoilage and softening.

Most essential oils (EOs) derived from plants are generally recognized as safe (GRAS), and they contain a variety of valuable bioactive substances with significant potential for use in the food industry to prevent food spoilage [15,16]. However, the inhibition of different active components varies, primarily depending on the types of active components in the EOs and the test strains [17,18,19]. D-limonene is a natural terpene commonly found in citrus EOs [20,21]. D-limonene can effectively inhibit foodborne and opportunistic pathogenic bacteria, including *Candida albicans*, *Staphylococcus aureus*, *Escherichia coli*, and *Klebsiella pneumoniae* [22,23,24]. Especially, D-limonene is considered the most promising monoterpene against unicellular fungi [24,25,26]. A recent study has revealed that 20 μL/mL D-limonene damages *Candida tropicalis* cell wall and membrane synthesis, interfering with cell membrane permeability and integrity [25]. Exposing *Listeria monocytogenes* to 20 μL/mL, D-limonene decreases the respiratory chain I–V complex activity, which is associated with oxidative phosphorylation. This interference affects respiratory function and energy metabolism, ultimately resulting in cell death [27]. However, no studies are available regarding the inhibitory effect of D-limonene on *Pichia kluyveri* during SCP film formation, while the mechanisms behind the D-limonene dysregulation of the phenotypic parameters in *P. kluyveri* remain unclear. 

Therefore, this study evaluated the gene transcription and metabolic level changes of *P. kluyveri* at different D-limonene treatment concentrations, including cell wall and membrane biosynthesis, mitochondrial oxidative phosphorylation, intracellular glycolysis, and tricarboxylic acid (TCA) cycling. Additionally, the cellular viability, cell morphology, ATP content, and endogenous reactive oxygen species (ROS), as well as the hexokinase (HK) and isocitrate dehydrogenase (IDH) levels, were analyzed. This study aimed to elucidate the molecular mechanisms behind the D-limonene inhibition of *P. kluyveri* and provide a reliable theoretical reference for preventing and controlling *P. kluyveri* film formation in SCPs.

## 2. Results

### 2.1. The Antifungal Activity of D-Limonene

**Figure 1:**
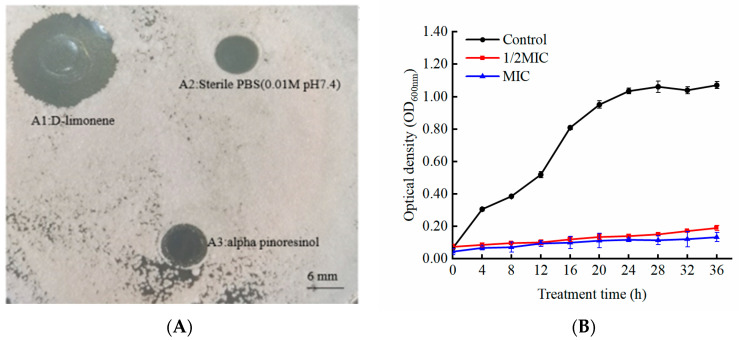
(**A**) The MIC of D-limonene. (**B**) The OD600 nm of *P. kluyveri* Y-11519 in the presence and absence of D-limonene at different concentrations.

The Oxford cup inhibition circle results indicated that D-limonene MIC of 20 μL/mL inhibited *P. kluyveri* Y-11519 (Figure 1A), showing significantly higher antifungal activity than the α-pinene solution (Figure 1(A3)). The *P. kluyveri* Y-11519 time-kill was evaluated at different D-limonene concentrations within 36 h to further analyze its antifungal activity. The fungal density of *P. kluyveri* Y-11519 in the untreated group increased rapidly after 4 h (Figure 1B). D-limonene treatment at a 1/2 MIC level significantly decreased the fungal density at 12 h, while *P. kluyveri* Y-11519 growth resumed from 14 to 36 h. Contrarily, an MIC completely inhibited *P. kluyveri* Y-11519 growth. In conclusion, a higher D-limonene concentration was more effective in inhibiting *P. kluyveri* Y-11519 proliferation.

### 2.2. The Effect of D-Limonene on the Cell Wall and Membrane

**Figure 2:**
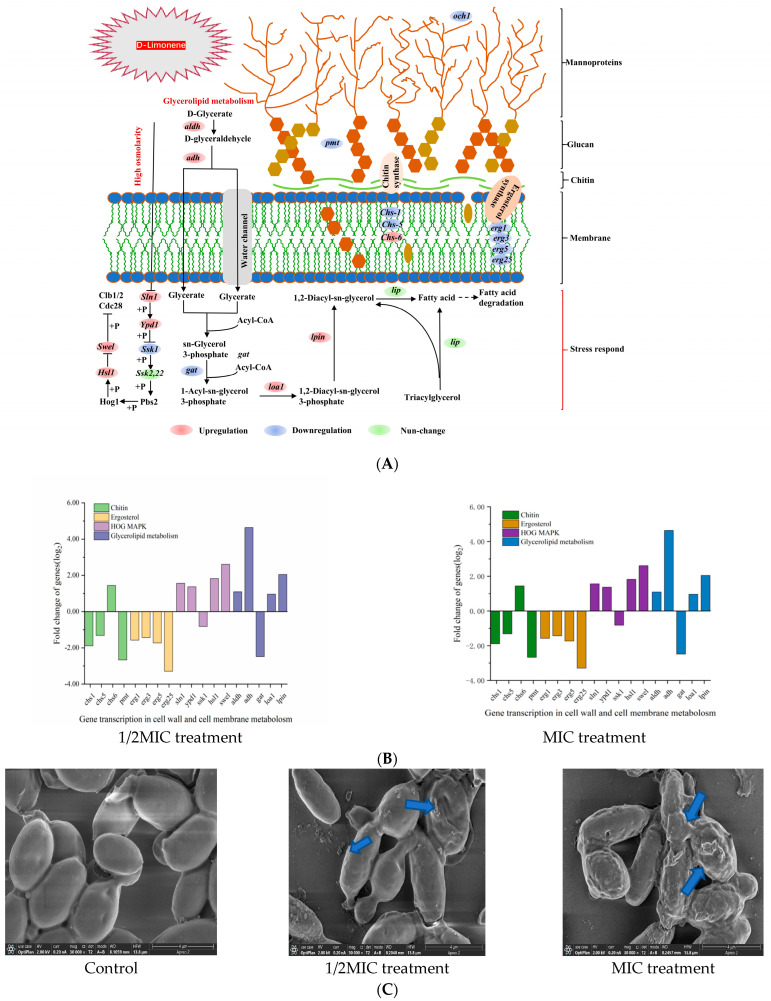

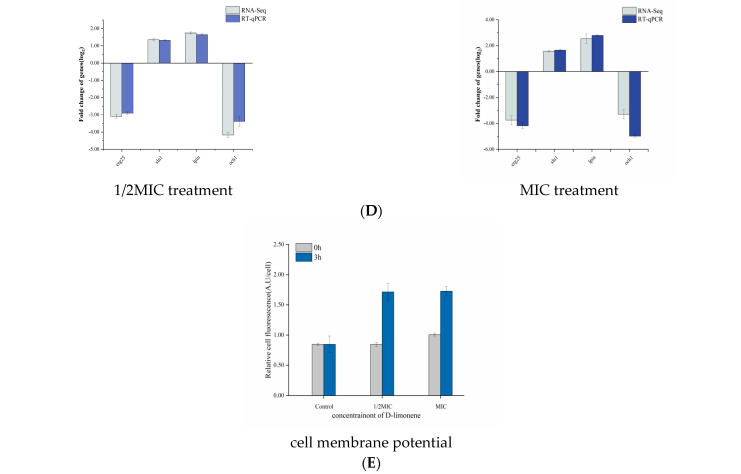
(**A**) A schematic diagram of the metabolic pathway of the cell wall and membrane components. (**B**) The effect of D-limonene on the gene transcription of cell wall and membrane metabolism. (**C**) The SEM images of *P. kluyveri* Y-11519, magnification 30,000×. (**D**) The differentially expressed genes in the cell wall and membranes were verified via RT-qPCR analysis. (**E**) The cell membrane potential of *P. kluyveri* Y-11519 treated with D-limonene for 3 h.

The fungal cell wall comprised glycoproteins, chitin, and β-glucans, conferring structural rigidity and elasticity to the cells. The outer layer consisted of mannoglycoprotein and dextran, forming a peptidoglycan network that determined cellular porosity [28] (Figure 2A). At 1/2MIC and 1 MIC D-limonene, the *chs-1* and *chs-5 P. kluyveri* Y-11519 genes responsible for encoding chitin synthase were down-regulated by 0.96–1.89 log_2_ and 1.32–2.49 log_2_, while the *chs-6* gene was up-regulated by 1–1.44 log_2_. The down-regulation of the *pmt* phosphomannose transferase gene and *och1* mannosyltransferase gene increased the cell wall porosity and chitin synthesis alterations (Figure 2B). Moreover, *och1* gene down-regulation was conducive to N-link mannosylation pathway disorder, hindering biofilm formation [29,30]. Exposing the cell wall to environmental stress activated the high-osmolarity glycerol (HOG) signaling pathway [31]. The key HOG-MAPK, *Sln1*, and *Ypd1* genes were up-regulated at 1/2MIC and 1 MIC of D-limonene concentrations, while *Ssk1* was down-regulated. The cell morphology showed contraction and adhesion, while the cell size and elasticity declined (Figure 2C). Since *Sln1* represented an intracellular histidine-encoding gene that affected HOG-MAPK pathway expression [32], it impacted *P. kluyveri* Y-11519 survival. RT-qPCR verified that *Sln1* was up-regulated 1.32–1.65 log_2_ fold compared with the control group (Figure 2D).

D-limonene also affected the cell membrane composition (Figure 2A) and down-regulated *erg1*, *erg3*, *erg5*, and *erg25* gene transcription. These changes in the encoding genes disrupted ergosterol synthesis, altering the cell permeability [33]. Glycerophospholipids constituted the main backbone of the cell membrane [34], while 1/2MIC D-limonene disrupted glycerophospholipid biosynthesis by down-regulating the *gat* gene 2.45 log_2_ fold and up-regulating the *loal* gene 1.02 log_2_ fold. Furthermore, phosphatidic acid phosphorylase (PAP) catalyzed 1,2-Diacyl-sn-glycerol-3-phosphate to produce 1,2-diacyl-sn-glycerol [35], showing that the *lpin* gene responsible for encoding phosphatidic acid phosphorylase was up-regulated (Figure 2B). PAP activity affected cell wall integrity, cell cycle death, oxidative stress, and fatty acid anabolism protein expression [36]. Moreover, 1,2-diacyl-glycerol helped regulate the cellular stimulus–response mechanism and controlled cellular biological activities, forming the premise for phospholipid and triglyceride synthesis in cells [37]. RT-qPCR confirmed a 2.90–4.19 log_2_ fold *erg25* gene down-regulation compared with the control group, while the *lpin* gene was up-regulated 1.65–2.79 log_2_ fold (Figure 2D). We used the lipophilic anionic fluorescent dye DiBAC4(3) to monitor changes in the membrane potential of *P. kluyveri* Y-11519. Under normal conditions, the cell membrane maintained a negative charge on the inside and a positive charge on the outside, sustained by ion channels and ion pumps on the membrane [38]. When the negative charge inside the cell membrane decreased, the dye entered the depolarized cells and bound to intracellular proteins, resulting in enhanced fluorescence. Conversely, in a hyperpolarized state, fluorescence decreased [39,40]. As shown in Figure 2E, a clear dose-dependent trend in fluorescence intensity increase was observed. These results suggested that D-limonene induced changes in the cell membrane composition and stability, altering membrane permeability and causing depolarization.

### 2.3. The Effect of D-Limonene on the Oxidative Phosphorylation Pathway

**Figure 3:**
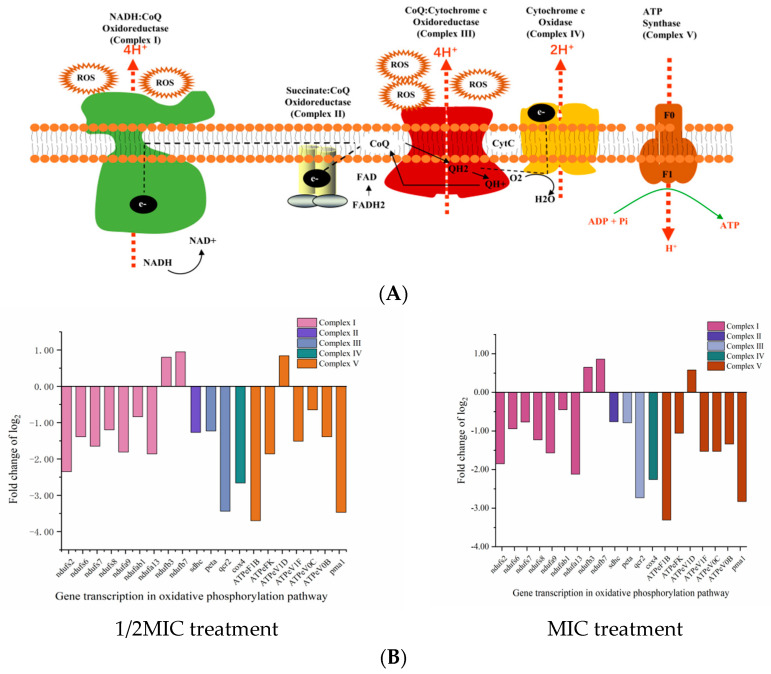

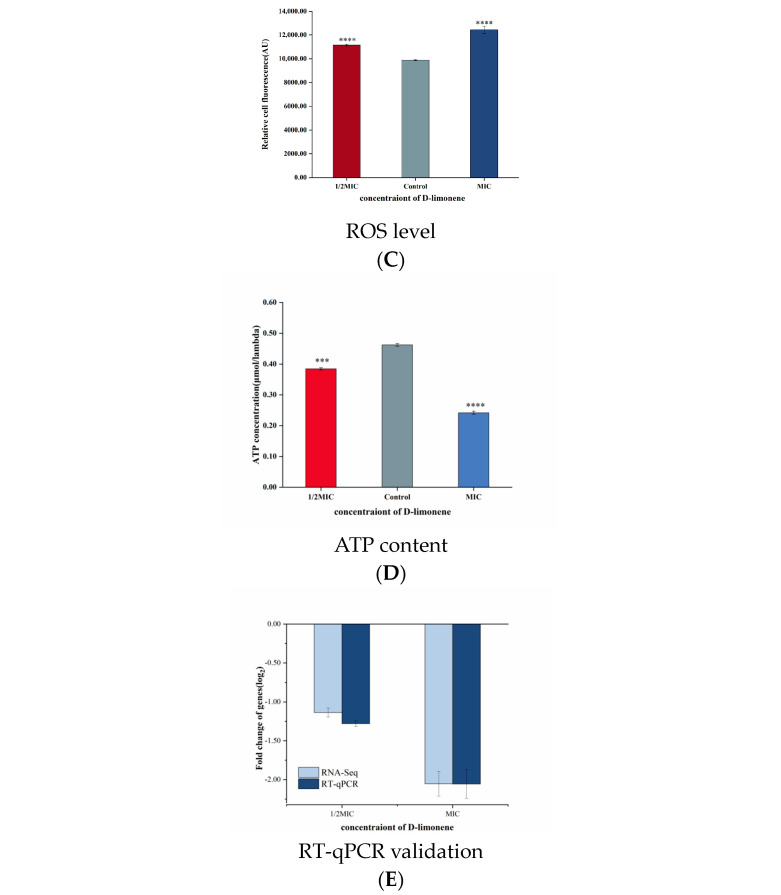
(**A**) A schematic diagram of the oxidative phosphorylation metabolic pathway. (**B**) The effect of D-limonene on the oxidative phosphorylation gene transcription in *P. kluyveri* Y-11519. (**C**) The endogenous ROS in *P. kluyveri* Y-11519 in the presence and absence of D-limonene at different concentrations (**** *p* < 0.0001). (**D**) The mitochondrial ATP concentration in *P. kluyveri* Y-11519 in the presence and absence of D-limonene at different concentrations (*** *p* < 0.001; **** *p* < 0.0001). (**E**) The *ATPeFK* gene expression was verified via RT-qPCR analysis.

Oxidative phosphorylation represents the main pathway of ATP and ROS production and is catalyzed by five mitochondrial complexes (I–V) [41] (Figure 3A). Dysfunction of either can lead to cellular homeostasis and oxidative stress [25,42]. Treating *P. kluyveri* Y-11519 with 1/2MIC and MIC D-limonene for 3 h up-regulated two mitochondrial complex I genes (*ndufb3* and *ndufb7*) and ATP synthase (*ATPeV1D*). Contrarily, seven genes (ndufs2, ndufs6, ndufs7, ndufs8, ndufa9, ndufab1, and ndufa13) encoding NADH dehydrogenase complex I, one gene (*sdhc*) encoding succinate dehydrogenase complex II, two genes (*peta* and *qcr2*) encoding cytochrome C reductase complex III, and one gene (*cox4*) encoding cytochrome C oxidase complex IV, as well as the ATP synthase genes (*ATPeF1B*, *ATPeFK*, *ATPeV1F*, *ATPeV0C*, *ATPeV0B*, and *pma1*), were down-regulated (Figure 3B). Alterations in the genes encoding mitochondrial complex I, complex II, and complex III increased the ROS level [41]. The *P. kluyveri* Y-11519 treated with MIC D-limonene showed a higher fluorescence value, displaying an ROS MFI increase of 20.43% (Figure 3C). ROS accumulation can lead to mitochondrial dysfunction, blocking electron transport and inhibiting proton pumping in the respiratory chain to reduce the ATP content [43] (Figure 3D). The results of this study were consistent with previous findings regarding the fungal inhibitory effect of essential oil and its ingredients [44,45,46]. ATP synthase produces cellular ATP by harnessing the energy stored in a transmembrane ion gradient [47], significantly impacting ATP production. The synthesis of ATP by F-type ATP synthase depends strictly on the transmembrane voltage [48]. To evaluate the gene expression changes, the F-type H+-transporting ATPase subunit (*ATPeFK*), responsible for encoding ATP synthase, was assessed using qRT-PCR. The results showed a 1.28- to 2.06-fold *ATPeFK* gene down-regulation compared with the control group (Figure 3E).

### 2.4. The Effect of D-Limonene on the Glycolytic Pathway

**Figure 4:**
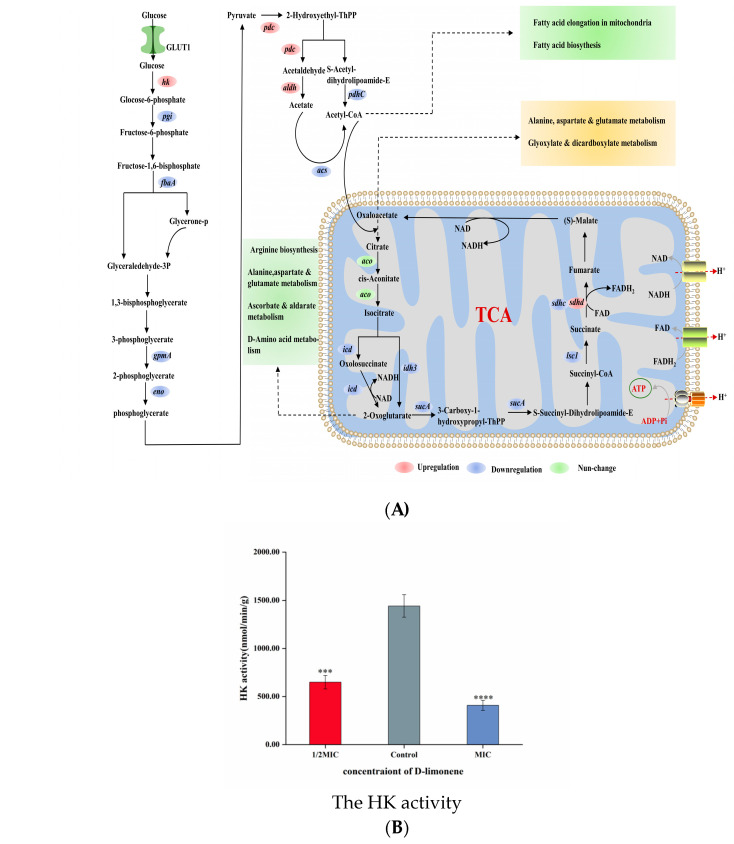

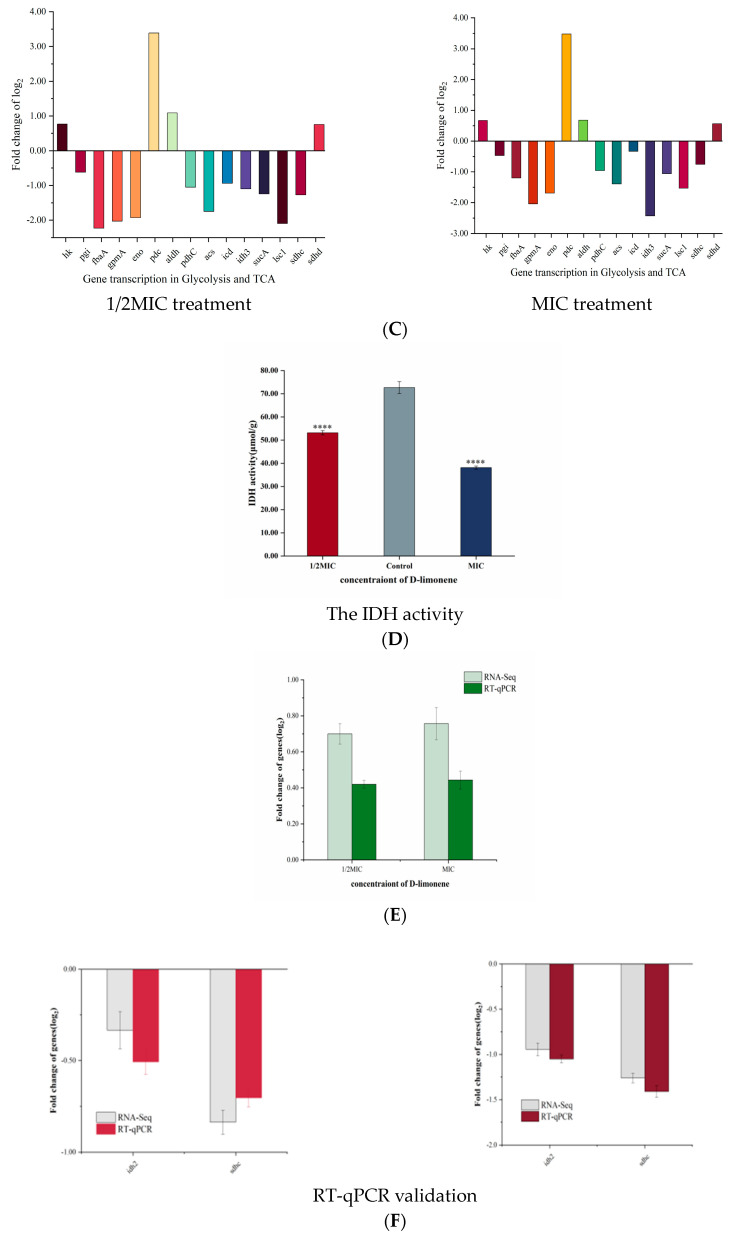
(**A**) A schematic diagram of the glycolysis and TCA cycle pathways. (**B**) The HK activity in *P. kluyveri* Y-11519 in the presence and absence of D-limonene at different concentrations (*** *p* < 0.001; **** *p* < 0.0001). (**C**) The effect of D-limonene on the glycolysis and TCA cycle gene transcription in *P. kluyveri* Y-11519. (**D**) The IDH activity in *P. kluyveri* Y-11519 in the presence and absence of D-limonene at different concentrations (**** *p* < 0.0001). (**E**) The *hk* gene expression was verified via RT-qPCR analysis. (**F**) The differentially expressed genes during the TCA cycle were verified via RT-qPCR analysis.

Glycolysis is considered the oldest energy metabolic pathway in microorganisms, representing a series of reactions in which glucose is converted to pyruvate in anaerobic conditions while also producing ATP and NADH [49]. HK is a key enzyme in the first step of glycolysis, catalyzing glucose phosphorylation and playing a crucial role during the preparatory phase [25] (Figure 4A). After treatment with 1/2MIC and 1MIC D-limonene, the HK activity decreased by 54.96% and 71.68% (Figure 4B). However, apart from the up-regulation of the *hk* gene, the expression levels of downstream genes related to the glycolytic pathway were all down-regulated (Figure 4E). The *pgi* gene responsible for encoding phosphoglucose isomerase was down-regulated 0.65-fold, while the glyceraldehyde-3-phosphate-encoding *fbaA* gene was down-regulated 0.21 log_2_ fold, after MIC D-limonene treatment. The *pgi* and *fbaA* genes were down-regulated 0.72- and 0.25-fold, respectively. During the ATP storage phase, the *gpmA* gene responsible for encoding phosphoglycerate mutase was down-regulated 0.25 log_2_ fold, while the enolase-encoding *eno* displayed a 0.26 log_2_ fold down-regulation. MIC D-limonene treatment reduced the gene change multiples to 0.25 and 0.31, respectively (Figure 4C). The intermediate glycolytic product, fructose-6-phosphate, is a crucial prerequisite for fungal cell wall biosynthesis and is derived from glucose-6-phosphate via phosphoglucose isomerase. The *pgi* gene responsible for encoding phosphoglucose isomerase inhibited *Aspergillus fumigatus* growth, while enolase, a key glycolytic enzyme involved in carbon metabolism, significantly impacted cell growth [50]. These transcriptomic results suggest that D-limonene reduced key enzyme gene expression in downstream pathways by inhibiting HK activity. RT-qPCR confirmed a 0.42–0.44 log_2_ fold *hk* gene up-regulation compared with the control group (Figure 4E).

### 2.5. The Effect of D-Limonene on the TCA Cycle

The TCA cycle plays a crucial role in the cellular metabolism of aerobic fungal organisms, providing energy by oxidizing and breaking down carbohydrates, fats, and amino acids and supplying intermediate products for the synthesis of biomolecules such as lipids and amino acids [51]. The TCA cycle involves a series of biochemical reactions inside the cell, where carbohydrates are broken down into pyruvate via the glycolytic pathway. The pyruvic acid enters the mitochondria and is oxidized to acetyl-CoA, which represents the starting point of the TCA cycle [52] (Figure 4A). After 1/2MIC D-limonene treatment, the *pdc* gene responsible for encoding pyruvate decarboxylase and the aldehyde dehydrogenase-encoding *aldh* were up-regulated 1.09- and 3.39 log_2_-fold, respectively. The *pdhc* gene responsible for encoding the pyruvate decarboxylase complex and the acetyl-CoA synthetase-encoding *acs* gene were down-regulated 1.05 and 1.75 log_2_ fold, respectively. The MIC D-limonene caused a 3.48- and 0.68 log_2_-fold *pdc* and *aldh* up-regulation, while *pdhC* and *acs* were down-regulated 0.96- and 1.39-fold (Figure 4C). During the TCA cycle enzyme catalyzation reactions, IDH, 2-oxyglutaric acid, succinyl-CoA ligase, and succinate dehydrogenase were encoded by the *idh*, *sucA*, *lsc1*, and *sdhc* genes, respectively. Furthermore, 1/2MIC and MIC D-limonene treatment inhibited gene expression, with only *sdhd* transcription displaying positive regulation. Isocitrate was converted to alpha-ketoglutarate by IDH, producing a low level of nicotinamide adenine dinucleotide cofactor [53]. Exposure to 1/2MIC and MIC D-limonene decreased the IDH activity in *P. kluyveri* Y-11519 by 26.39% and 47.2%, respectively, compared with the blank control (Figure 4D). The inhibitory effect became more obvious at a higher D-limonene concentration. In addition, succinate dehydrogenase catalyzed succinate conversion to fumaric acid, while FAD was converted to FADH_2_ [52]. D-limonene inhibited the TCA cycle in the *P. kluyveri* Y-11519 mitochondria, reducing the NADH and FADH_2_ substrate supply required for oxidative phosphorylation, impacting ATP production. RT-qPCR was conducted to verify the influence of D-limonene treatment on the *P. kluyveri* Y-11519 gene expression levels using the *idh2* and *sdhd* genes. All these genes were consistent with the transcriptomics results (Figure 4F).

## 3. Discussion

Whether produced on a household or industrial scale, SCPs are prone to spoilage by undesirable microorganisms during natural fermentation. Lemon peels are commonly used as a preservative in household pickles, while industrial production of SCP often employs methods such as increasing salt concentration and low-temperature storage to prevent membrane spoilage of fermented vegetables. However, wastewater pollution during desalination and uncontrollable conditions limit the development of industrial pickles. D-limonene, the main component of citrus essential oil, has potential as an antimicrobial agent, but its antimicrobial mechanism against *Pichia kluyveri* remains unclear. Therefore, this study is the first to elucidate the molecular mechanism by which D-limonene disrupts metabolic pathways and inhibits *P. kluyveri* Y-11519. Furthermore, our research may provide new insights into using D-limonene to solve other food spoilage issues caused by yeast. 

This study employed Oxford cup diffusion and liquid culture assays to verify the ability of D-limonene to inhibit *P. kluyveri* Y-11519. The scanning electron microscopy results showed that D-limonene damaged the *P. kluyveri* Y-11519 cell morphology, depressing the cell surface to cause content leakage. Previous studies have demonstrated that glucan, chitin, and mannoprotein, the main cell wall components, are often considered natural action target inhibitors [25]. Molecular docking confirmed that citral bonded to *Aspergillus niger* chitin synthase protein, damaging cell wall integrity [54]. In another study, paeonol reduced chitinase activity by down-regulating chitinase class III and class V genes [55]. Treating the *Geotrichum citri-aurantii* citrus pathogen with cinnamaldehyde down-regulated the *chs2* and *chs5* chitin-related genes and reduced the chitin content [56]. In the present study, D-limonene treatment down-regulated *chs-1* and *chs-5* and up-regulated *chs-6* (Figure 2B), while any changes in the gene encoding chitin synthase affected chitin synthesis. Mannoprotein is located in the outermost cell wall layer and represents the first location of fungal and host immune response [57]. Och1 is an initiating-specific α-1,6-mannosyltransferase crucial for outer mannose chain elongation. It affects cell porosity and regulates biofilm formation by influencing the mannosylation pathway. Pmt is a mannosyltransferase that participates in mannose residue transfer from phosphodolichol phosphate D-mannose to serine and threonine residues in proteins. *Pmt* gene mutations cause significant defects in cell morphology and integrity [58,59]. Both the *Och1* and *Pmt* expression levels were down-regulated in the experimental group. *P. kluyveri* Y-11519 activated the HOG signaling pathway in response to hyperosmotic pressure, with the HOG-MAPK kinase cascade representing the core element. Activated Hog1 regulates the cell cycle, protein translation, and gene expression [60,61]. Under the osmotic stress induced by D-limonene, Sln1 undergoes autophosphorylation at His, then transfers the phosphorylation moiety to Ssk1 via the intermediate Ypd1 protein. Phosphorylated Ssk1 inhibits redundant Ssk2 and Ssk22 MAPKKKs, promoting downstream Pbs2 MAPKK and Hog1 MAPK activation. Cdc28 kinase is a cyclin-dependent kinase (CDK) involved in cell cycle regulation. CDK forms complexes with cyclins to regulate progression through the different cell cycle stages [62]. Hsl1 is a Cdc28 kinase substrate that plays a role in coordinating cell cycle progression and cell division [60]. The MAPK1 pathway up-regulates the negative Swel regulatory kinase, activating *Hsl1* gene expression and Cdc28±Clb complex encoding. Swel kinase specifically inhibits the G2 phase via the Tyr19 phosphorylation of Cdc28, delaying the onset of mitosis [63]. Research has shown that treatment with perilla oil, which is associated with cell cycle-related genes, down-regulates and inhibits *Aspergillus* spore development [45]. Similar results were observed in several earlier studies, suggesting that terpenoid compounds disrupted the cell cycle process and induced cell cycle arrest [64,65].

Ergosterol has attracted extensive attention as a target of natural bacteriostatic agents and is essential for maintaining cell function [66]. Its synthesis involves a complex metabolic process that requires the participation of multiple enzymes, starting with mevalonic acid conversion to lanosterol, followed by lanosterol cyclization. The lanosterol undergoes multiple metabolic transformations to produce ergosterol [67]. The current study indicated that the *erg1* gene encoded lanosterol 14 α-demethylase, the *erg3* gene encoded sterol C-5 desaturase, the *erg5* gene encoded sterol C-22 desaturase, and the *erg25* gene encoded sterol C-4 methyl oxidase. All these genes were down-regulated, which inhibited ergosterol synthesis. Similar research findings showed that citral affected *Penicillium digitatum* by disrupting the expression levels of the five genes (*ERG7*, *ERG11*, *ERG6*, *ERG3*, and *ERG5*) involved in the ergosterol biosynthesis pathway [68]. Similarly, the lipophilic nature of D-limonene allowed it to bind to the lipid membrane, which reduced ergosterol synthesis while increasing membrane fluidity and permeability and cellular content leakage (Figure 2C). D-limonene treatment changed the expression levels of the genes related to the glycerophospholipid synthesis pathway in *P. kluyveri* Y-11519, down-regulating *gat* and up-regulating *loa1* and *lpin*. This suggests changes in the cell membrane composition, leading to cell membrane instability and depolarization (Figure 2E).

Living cells use ATP to organize and manipulate macromolecule synthesis and polar membrane molecule or ion transport in cells. Oxidative phosphorylation (OXPHOS), which occurs in the mitochondria of aerobic fungal cells, is the main pathway for ATP synthesis and plays a crucial role in maintaining cellular ATP levels. The substrate-level phosphorylation in the glycolysis pathway and TCA cycle supplements ATP synthesis, directly generating ATP and NADH without the electron transport chain. Therefore, blocking or restraining oxidative phosphorylation can effectively decrease ATP concentrations in the cell. As shown in Figure 3D, *P. kluyveri* Y-11519 displayed increased ROS levels (Figure 3C), possibly due to changes in the genes responsible for encoding mitochondrial complexes I, II, and III. ROS accumulation caused oxidative stress, leading to mitochondrial dysfunction [41,69,70]. Furthermore, lower IDH activity decreased the TCA cycle level and the NADH and FADH_2_ substrate supply required for oxidative phosphorylation. Similarly, the activity of hexokinase, the first key enzyme during the glycolysis process, was significantly lower (Figure 4C). However, apart from the up-regulation of the *hk* gene, the expression levels of downstream genes related to the glycolytic pathway were all down-regulated (Figure 4E). This regulation is actually controlled by cell metabolism and gene expression. Hexokinase is typically inhibited by glucose-6-phosphate. Under D-limonene stress, when the activity of phosphofructokinase is inhibited, glucose-6-phosphate accumulates, affecting the glucose phosphorylation process. This slows down glycolysis and triggers a stress response in the cell, leading to the up-regulation of the *hk* gene. The changes in glycolytic intermediates and the TCA cycle impact the metabolic regulation of other cellular pathways [51]. For example, lipid metabolism disturbances change the membrane lipid content, resulting in secondary membrane damage [46,71], while carbohydrate metabolism affects the cell wall polysaccharide content [72]. Therefore, D-limonene interferes with oxidative phosphorylation, the TCA cycle, and glycolytic pathways in *P. kluyveri* Y-11519, hindering energy metabolism in the cells and ultimately causing cell death.

## 4. Materials and Methods

### 4.1. Strains and Reagents

The *Pichia kluyveri* Y-11519 strain was isolated from membrane-spoiled Sichuan pickles and obtained from the Sichuan Fruit and Vegetable Preservation Laboratory at Xihua University (Chengdu, China). The yeast extract peptone dextrose (YPD) medium was purchased from Hangzhou Microbiological Reagent Co., Ltd. (Hangzhou, China). The *P. kluyveri* Y-11519 was serially scribed on YPD plates up to the third generation to obtain single colonies and stored at 4 °C. It was activated and cultured to the logarithmic growth stage, after which the cell suspension was adjusted with sterile saline to a concentration of about 1 × 10^7^ CFU/mL for subsequent use. The D-limonene reagent (HPLC purity ≥ 98%, Beijing Quantitative Spectroscopy Technology Co., Ltd. (Beijing, China)) was diluted to 0 μL/mL, 5 μL/mL, 10 μL/mL, 20 μL/mL, 40 μL/mL, and 60 μL/mL with phosphate-buffered saline (PBS, 0.01 mol/mL, pH 7.2); passed through a 0.22 μm microporous filter membrane; and stored at −20 °C.

### 4.2. Antifungal Activity 

Here, 100 μL of the *P. kluyveri* Y-11519 suspension at about 1 × 10^7^ CFU/mL was evenly spread on a sterile YPD plate, after which a sterilized Oxford cup was placed on the test plate using forceps. Then, 100 μL of the different D-limonene concentrations (0 μL/mL, 5 μL/mL, 10 μL/mL, 20 μL/mL, 40 μL/mL, and 60 μL/mL) was added to the Oxford cup [24,73], while sterile PBS (0.01 mol/mL, pH 7.2) was used as a negative control. Since research confirmed the sensitivity of uncommon yeast to α-pinene [74], this study used a 20 μL/mL α-pinene solution as a positive control. After static incubation for 24 h at 25 °C, the lowest D-limonene concentration with an inhibition circle (cm) exceeding 10 cm around the Oxford cup was used as the minimum inhibitory concentration (MIC) [75]. The time-kill curve of the D-limonene against *P. kluyveri* Y-11519 was measured every 4 h according to the fungal density at a wavelength of 600 nm. Then, 100 μL of the *P. kluyveri* suspension was inoculated during the logarithmic growth phase, and the 1/2MIC and MIC of D-limonene were added and incubated for 36 h at 25 °C in fresh YPD liquid medium. The control group was not treated with D-limonene. The growth curve was plotted as the absorbance at 600 nm against the time interval.

### 4.3. Cell Morphology

Scanning electron microscopy was used to determine the impact of D-limonene on the *P. kluyveri* Y-11519 morphology at the 1/2MIC and MIC levels [76], while the blank control contained no added D-limonene. Next, 200 μL of the respective *P. kluyveri* Y-11519 cell suspensions were inoculated into 10 mL YPD with 1/2MIC and MIC D-limonene. The mixtures were incubated at 25 °C for 3 h, centrifuged at 6000 rpm for 10 min, fixed with a 2.5% glutaraldehyde solution for 3 h, centrifuged for 10 min, and washed three times with sterile PBS (0.01 mol/mL, pH 7.2). The samples were placed on slides; rinsed sequentially with 20%, 40%, 60%, 80%, and 100% ethanol concentrations; exposed to graded dehydration; and wrapped in taped cling film. The slides were pre-frozen at −20 °C for 12 h and freeze-dried for 24 h, after which they were sprayed with gold and photographed microscopically.

### 4.4. Cell Membrane Potential

The cell membrane potential was determined using DiBAC_4_(3) voltage-sensitive fluorescent dye. The logarithmic-growth-phase *P. kluyveri* Y-11519 was adjusted to 1 × 10^7^ CFU/mL and incubated with 1/2MIC and MIC of D-limonene in the YPD at 25 °C for 4 h. The cells were collected and washed three times with sterile PBS (0.01 mol/mL, pH 7.2). DiBAC_4_(3) dissolved in sterile PBS (0.01 mol/L, pH 7.2) was added to the cell suspension at a final concentration of 10 μg/mL and incubated for 20 min at room temperature in the dark. Cells without D-limonene treatment were used as a blank control. The fluorescence intensity (MFI) was measured at excitation and emission wavelengths of 490 nm and 540 nm, respectively.

### 4.5. Mitochondrial Adenosine Triphosphate (ATP) Content

Here, 250 μL of the *P. kluyveri* Y-11519 cell suspension was inoculated into 5 mL YPD containing 1/2MIC and MIC of D-limonene and incubated at 25 °C for 3 h. Cells without D-limonene were used as a blank control. After centrifugation at 4 °C and 6000 rpm for 10 min, the cells were washed three times with sterile PBS (0.01 mol/mL, pH 7.2), after which the supernatant was discarded to obtain the *P. kluyveri* Y-11519 cells. The changes in the ATP content before and after treating the *P. kluyveri* Y-11519 with D-limonene were determined using an ATP kit (Nanjing Jiancheng Institute of Biological Engineering Co., Ltd., Nanjing, China).

### 4.6. Endogenous ROS

The ROS levels in the *P. kluyveri* Y-11519 cells were determined using 2′,7′-dichlorodihydrofluorescein diacetate (DCFH-DA) [20]. The *P. kluyveri* Y-11519 was exposed to D-limonene (1/2MIC and MIC) for 3 h at 25 °C in a shaker at 140 rpm, after which the cells were collected (6000 rpm, 10 min) and washed three times with sterile PBS (0.01 mol/mL, pH 7.2). The cells were resuspended in 1 mL of 10 μM DCFH-DA and incubated in the dark for 30 min at 25 °C. The sample was mixed upside down at 5 min intervals during incubation to ensure full contact between the probe and the cells, followed by centrifugation, washing, and resuspension at a volume of 1 mL. Then, 100 μL of the cells was placed in a black 96-well plate, and each concentration gradient was assessed using three parallel samples at excitation and emission wavelengths of 488 nm and 525 nm, respectively. PBS was used as a blank control.

### 4.7. The HK Activity

The *P. kluyveri* Y-11519 cells in the logarithmic growth phase were collected, supplemented with 1/2MIC and 1 MIC of D-limonene, and incubated at 25 °C for 3 h while shaking. The cells were collected (6000 rpm for 10 min) and washed three times with sterile PBS (0.01 mol/mL, pH 7.2). An HK activity assay kit (Nanjing Jiancheng Institute of Biological Engineering Co., Ltd., Nanjing, China) was used for measurement at 340 nm.

### 4.8. The IDH Activity

The *P. kluyveri* Y-11519 cells in the logarithmic growth phase were collected, supplemented with 2MIC and 1 MIC of D-limonene, and incubated at 25 °C for 3 h while shaking. The cells were collected (6000 rpm for 10 min) and washed three times with sterile PBS (0.01 mol/mL, pH 7.2). An IDH activity assay kit ((Beijing Solarbio Technology Co., Ltd., Beijing, China) was used for measurement at 340 nm.

### 4.9. RNA Sequencing and RT-PCR Analysis

TRIzol reagent (Thermo Fisher Scientific Inc., Waltham, MA, USA) was used for RNA extraction from the *P. kluyveri* Y-11519 cells. Previously described methods were used to assess the RNA quality [25] and analyze the RNA-Seq and RT-PCR data [77]. TB Green Premix Ex Taq II was selected as the light dye during qRT-PCR, while the primers were designed using the https://www.ncbi.nlm.nih.gov/tools/primerblast/index.cgi?LINK_LOC=BlastHome (accessed on 8 March 2023) website. Table S1 shows the primer sequences used in this study. Furthermore, 18S rRNA genes were used as an internal reference, while the relative gene expression ploidy was calculated via 2^−∆∆CT^ [45].

### 4.10. Statistical Analysis

The experiment was repeated three times for each sample, and the data were expressed as the mean ± standard deviation. A *p* < 0.05 value was considered significant. The data were visualized using Origin 2021 (Origin 2021 pro Software, Northampton, MA, USA).

## 5. Conclusions

In summary, D-limonene interferes with cell wall and membrane biosynthesis, oxidative mitochondrial phosphorylation, intracellular glycolysis, and the TCA cycle in *P. kluyveri* Y-11519. This reduces cellular activity, alters cellular morphology, and decreases the ATP content and endogenous ROS accumulation. The HK and IDH activity declines significantly, causing cellular metabolism dysfunction. These findings help understand the molecular mechanism behind D-limonene *P. kluyveri* Y-11519 inhibition. Of course, the effects of D-limonene on other microorganisms in SCP, changes in physicochemical indexes, and overall flavor acceptability need to be further studied.

## Data Availability

The original contributions presented in the study are included in the article (and Supplementary Material), further inquiries can be directed to the corresponding authors.

## Supplementary Materials

The following supporting information can be downloaded at: https://www.mdpi.com/article/10.3390/molecules29153561/s1. Table S1: The primer sequences used in this study.
