# Supplementary material for: D-Limonene Inhibits Pichia kluyveri Y-11519 in Sichuan Pickles by Disrupting Metabolism
Source: Molecules. 2024 Jul 28;29(15):3561. doi: 10.3390/molecules29153561 (PMC11314558; doi:10.3390/molecules29153561)
Supplement: Supplementary file 1 [file molecules-29-03561-s001.zip › molecules-3067895-supplementary.pdf]

Supplementary Materials

D-Limonene Inhibits *Pichia kluyveri* Y-11519 in Sichuan Pickles by Disrupting Metabolism

Chaoyi Zeng [1,2,3,*], Yue Sun [1,3], Haoran Lin [1,3], Ziyu Li [1,3], Qing Zhang [1,3], Ting Cai [1,3], Wenliang Xiang [1,3], Jie Tang [1,2,3] and Patchanee Yasurin [2]

[1] College of Food and Bioengineering, Xihua University, Chengdu 610039, China; 17780770409@163.com (Y.S.); 212022095100016@stu.xhu.edu.cn (H.L.); catalpa_123@163.com (Z.L.); biozhangq@163.com (Q.Z.); caiting1124@mail.xhu.edu.cn (T.C.); biounicom@mail.xhu.edu.cn (W.X.); tangjie1225@mail.xhu.edu.cn (J.T.)
[2] Department of Food Biotechnology, Faculty of Biotechnology, Assumption University, Bangkok 10240, Thailand; patchaneeysr@au.edu
[3] Food Microbiology Key Laboratory of Sichuan Province, Xihua University, Chengdu 610039, China
* Correspondence: chaoyi@mail.xhu.edu.cn

Table S1. The primer sequences used in this study.

Gene name	Forward primer	Reverse primer
18s rRNA	TTGACTGAATTTTCAGCGCCAT	CTACACTGACGGAGCCAGCAAG
icd	AGGTGGTACCGTTTTCAGAGA	TCAGCATGAGCATGACGACC
hk	TGGGTACTTCCCACATCACG	TCCATCTGCACCTTCAACGG
erg25	AGGTGCAGGTACTGTTGGTT	CACCTGCCCAGAATGGTACA
sln1	TGGATGGTTTATCGGCTACTCG	TCCAGACATGCCAGCATCAA
sdhc	GGTGTTGGTCTTGCTGTTGG	GCTTCATGACCAGCATCCCA
ATPeFK	TCTGTTCCTCCACAAATTCTTGC	AGTTGCAGCCCATGGTTTTG
lpin	TGAGGAGGAGGTAGATTCCGA	TTGCCGCTTCCGATTGTCTA
och1	GGCAGATCCGGACAGAGATG	ACTGACAGGAACTCCTGGGT
